# Recurrent Intussusception in a Pediatric Patient: The Role of the Mesoappendix as a Potential Culprit

**DOI:** 10.7759/cureus.74695

**Published:** 2024-11-28

**Authors:** Antonio Santana Veliz, Danielle Passafiume, Brandon Zarnoth, Anand Majmudar, Ravikumar Hanumaiah

**Affiliations:** 1 Diagnostic Radiology, State University of New York Upstate Medical University, Syracuse, USA; 2 Pediatric Radiology, State University of New York Upstate Medical University, Syracuse, USA; 3 Radiology, State University of New York Upstate Medical University, Syracuse, USA

**Keywords:** abdominal pain in children, appendiceal intussusception, gastrointestinal disorders, mesoappendix, pediatric radiology, recurrent ileocolic intussusception, recurrent intussusception

## Abstract

Intussusception, a condition in which one part of the intestine telescopes into another, primarily affects children under 18 months of age. This case report details the radiologic findings in a six-year-old child with a long-standing history of recurrent ileocolic intussusception, who presented with abdominal pain and was diagnosed with intussusception-associated appendicitis. Following the fifth recurrence, the patient underwent laparoscopic reduction of the intussusception and appendectomy. Surgical findings revealed an inflamed appendix, prominent mesoappendix, and adhesions with mesenteric fat, which likely contributed to the recurrent episodes. This case underscores the importance of considering anatomical anomalies in the differential diagnosis to optimize management and prevent further recurrences.

## Introduction

Intussusception occurs when one part of the intestine slides into an adjacent segment, much like the sections of a telescope. It most commonly affects infants between four and nine months of age, with the incidence tapering off by around 18 months. Males are affected more frequently than females, and recurrence is relatively uncommon, occurring in only about 10% of pediatric cases [1].

This condition involves the telescoping of one bowel segment, along with its mesentery, into a neighboring section. It is one of the leading causes of intestinal obstruction in children under the age of three [2]. Timely diagnosis is crucial, with symptoms such as abdominal pain, vomiting, and irritability serving as key indicators.

When evaluating children with acute abdominal pain, radiologists should consider the possibility of ileocolic intussusception, particularly in cases involving anatomical variations [2]. A prominent mesoappendiceal curtain or adhesions may act as a lead point, and recognizing these variants can help guide management and prevent complications.

## Case presentation

A six-year-old child with a medical history significant of asthma and multiple previous episodes of ileocolic intussusception presented to the emergency department with a 20-hour history of abdominal pain. The patient had experienced four prior episodes of ileocolic intussusception, all successfully resolved with fluoroscopic hydrostatic reduction. At three years old, the patient had two episodes of intussusception within one month. At four years old, the patient had two additional episodes, occurring two months apart. Previous investigations revealed no clear causes, such as Meckel's diverticulum, inflammation, masses, or other identifiable leading points.

On presentation, the abdominal pain was described as intermittent, with sudden, intense episodes lasting 5-10 minutes. The patient's parents reported that the pain was similar in nature to his previous episodes of intussusception. The patient denied nausea, vomiting, diarrhea, fever, or chills at the time of evaluation. On physical examination, the patient was tender to palpation inferior to the umbilicus, but there were no signs of peritonitis, guarding, or hemodynamic instability.

**Figure 1:**
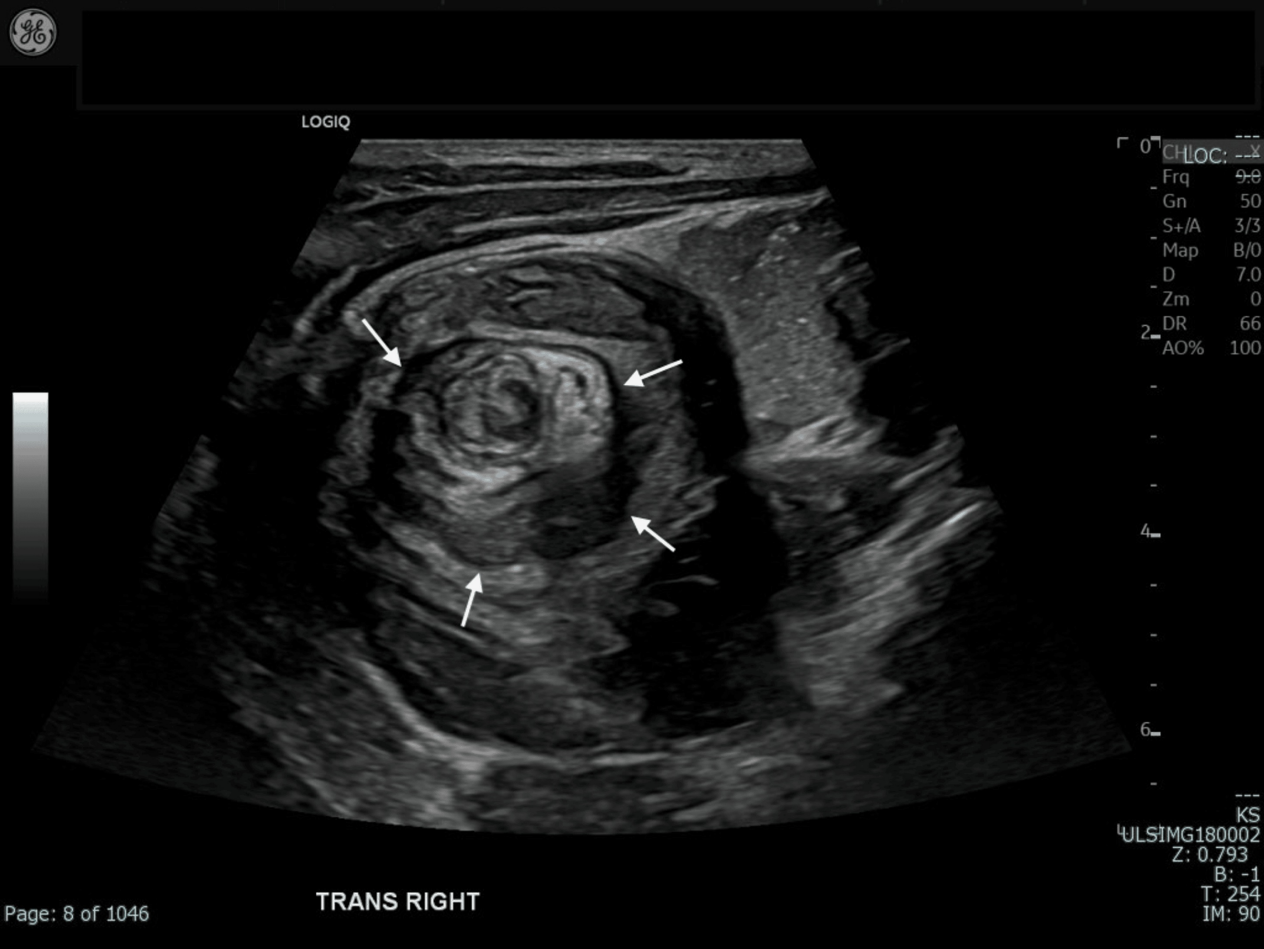
Target lesion and telescoping bowel loops indicative of intussusception (white arrows). Based on its size and location, the intussusception was categorized as ileocolic.

**Figure 2:**
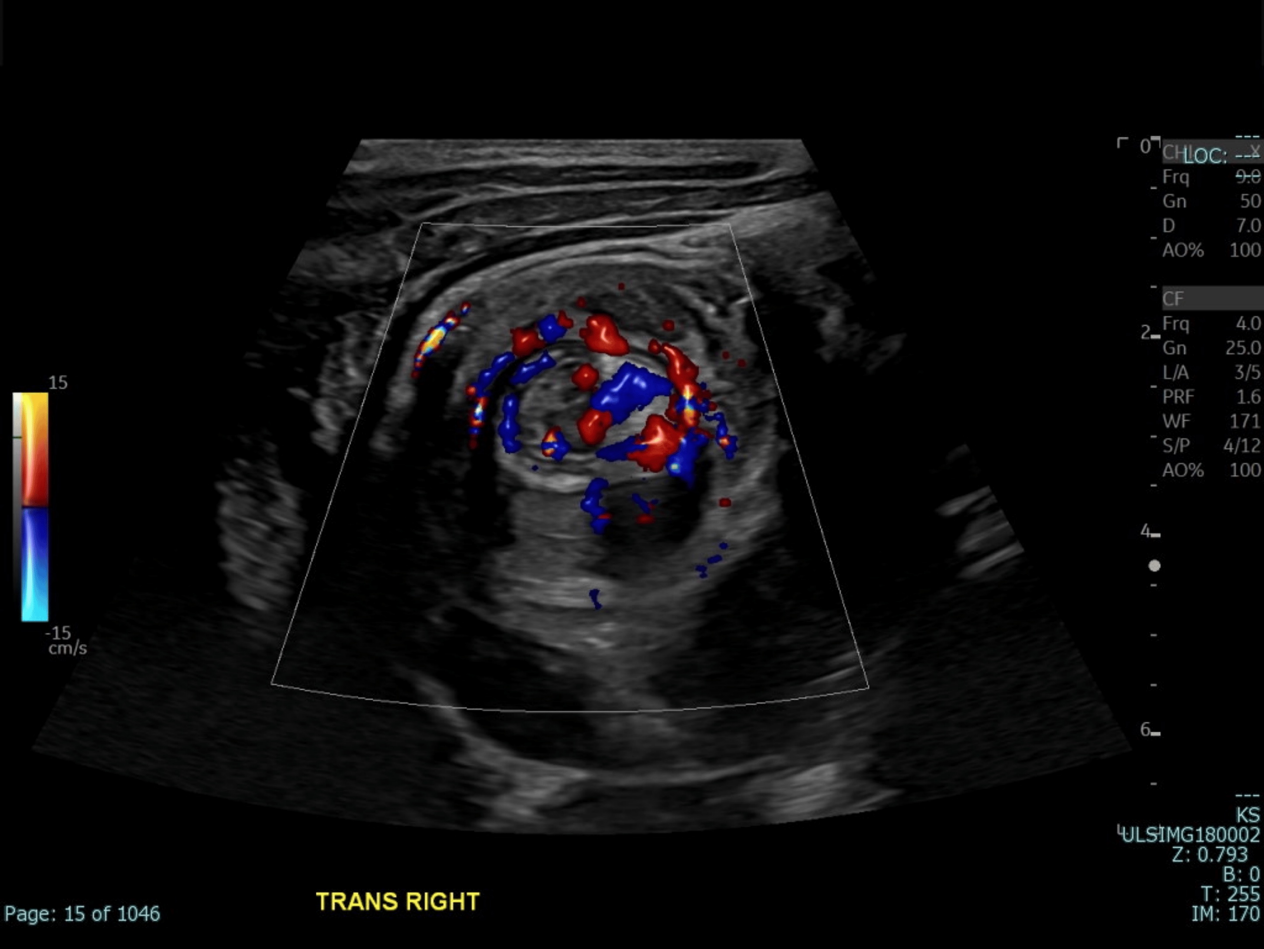
Color Doppler imaging reveals continuous blood flow within the ileocolic segment affected by the intussusception.

**Figure 3:**
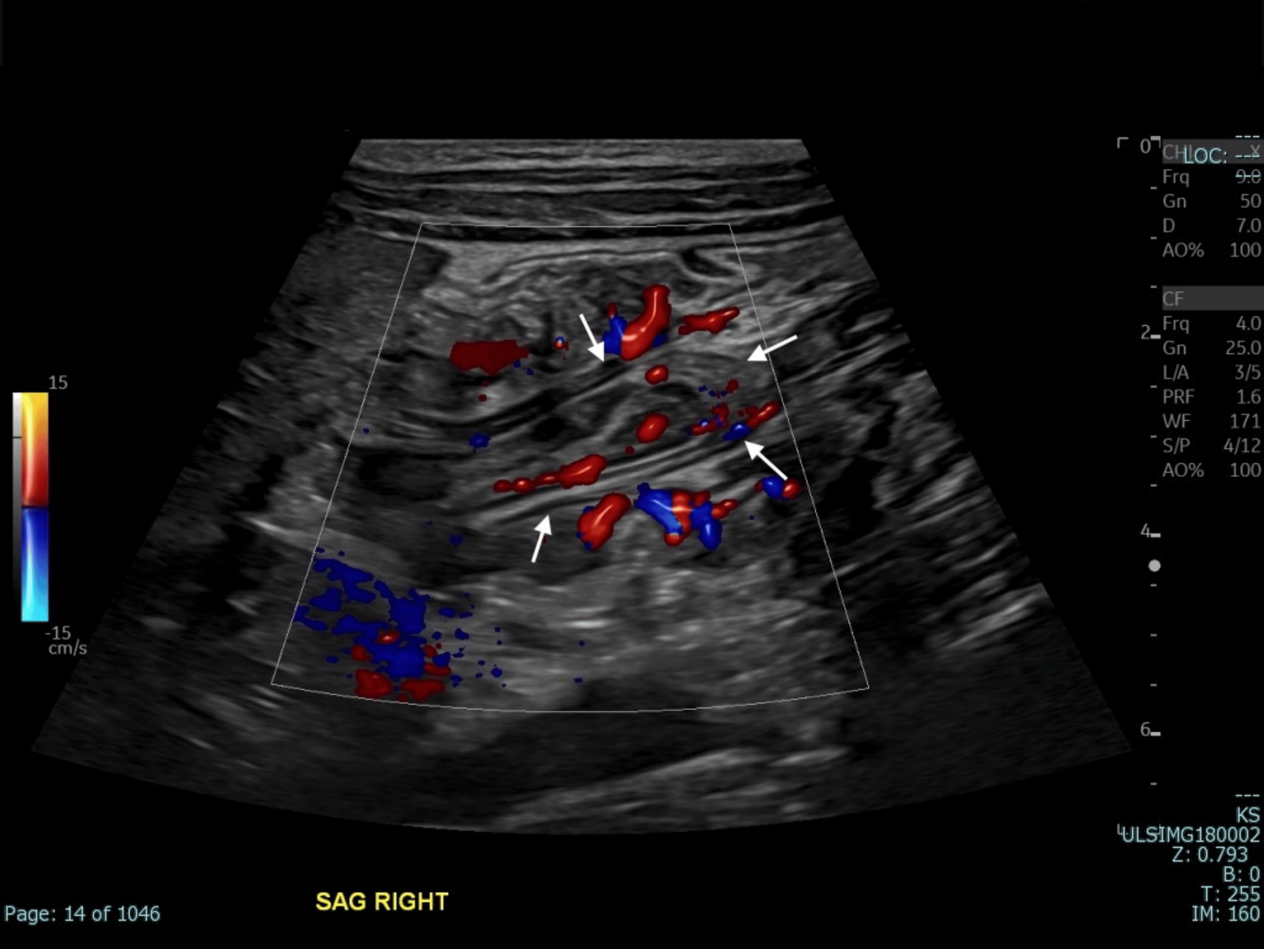
The appendix is thickened and inflamed with mild increased vascularity (white arrows).

**Figure 4:**
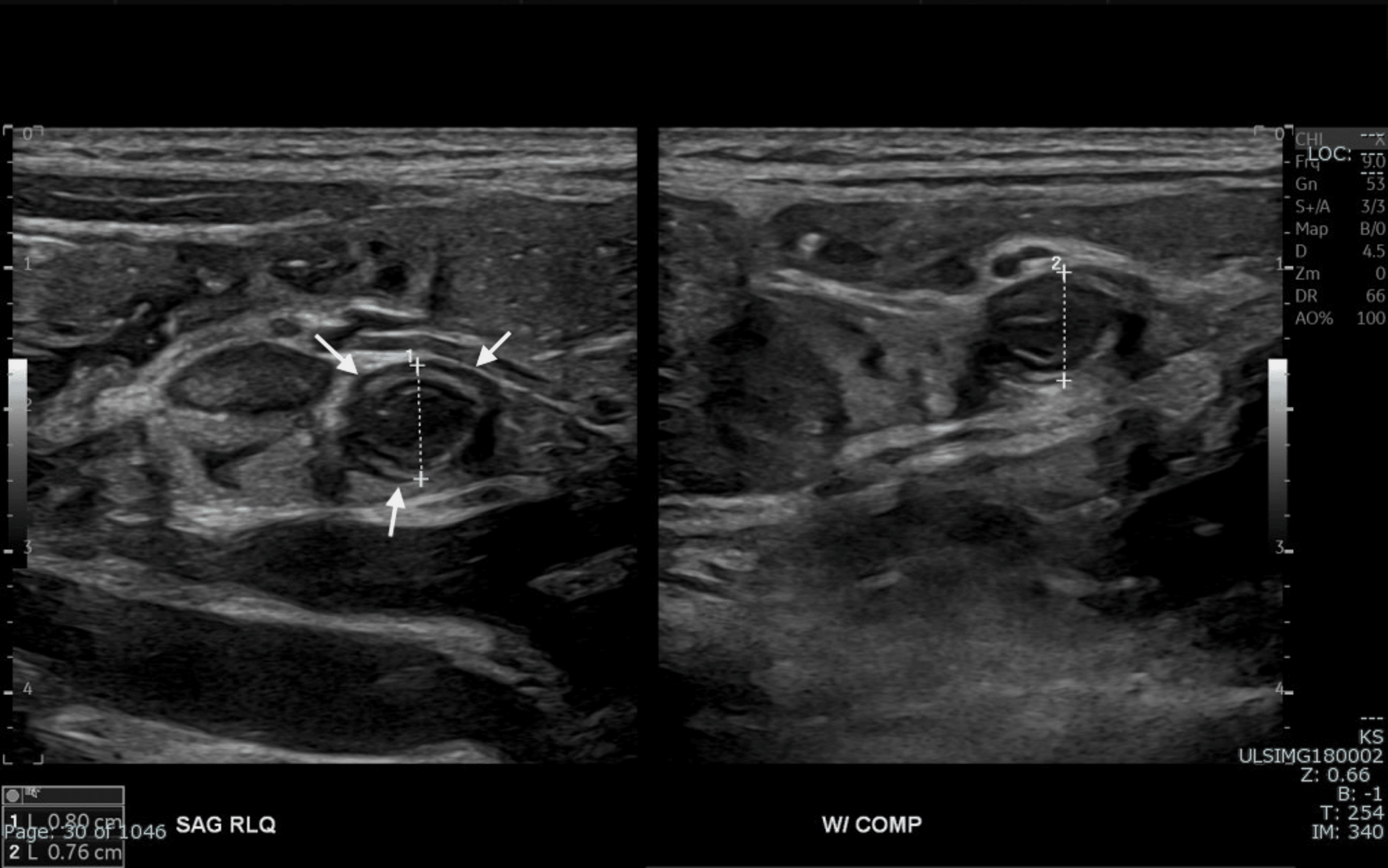
The distal part of the appendix shows dilatation and non-compressibility (white arrows).

**Figure 5:**
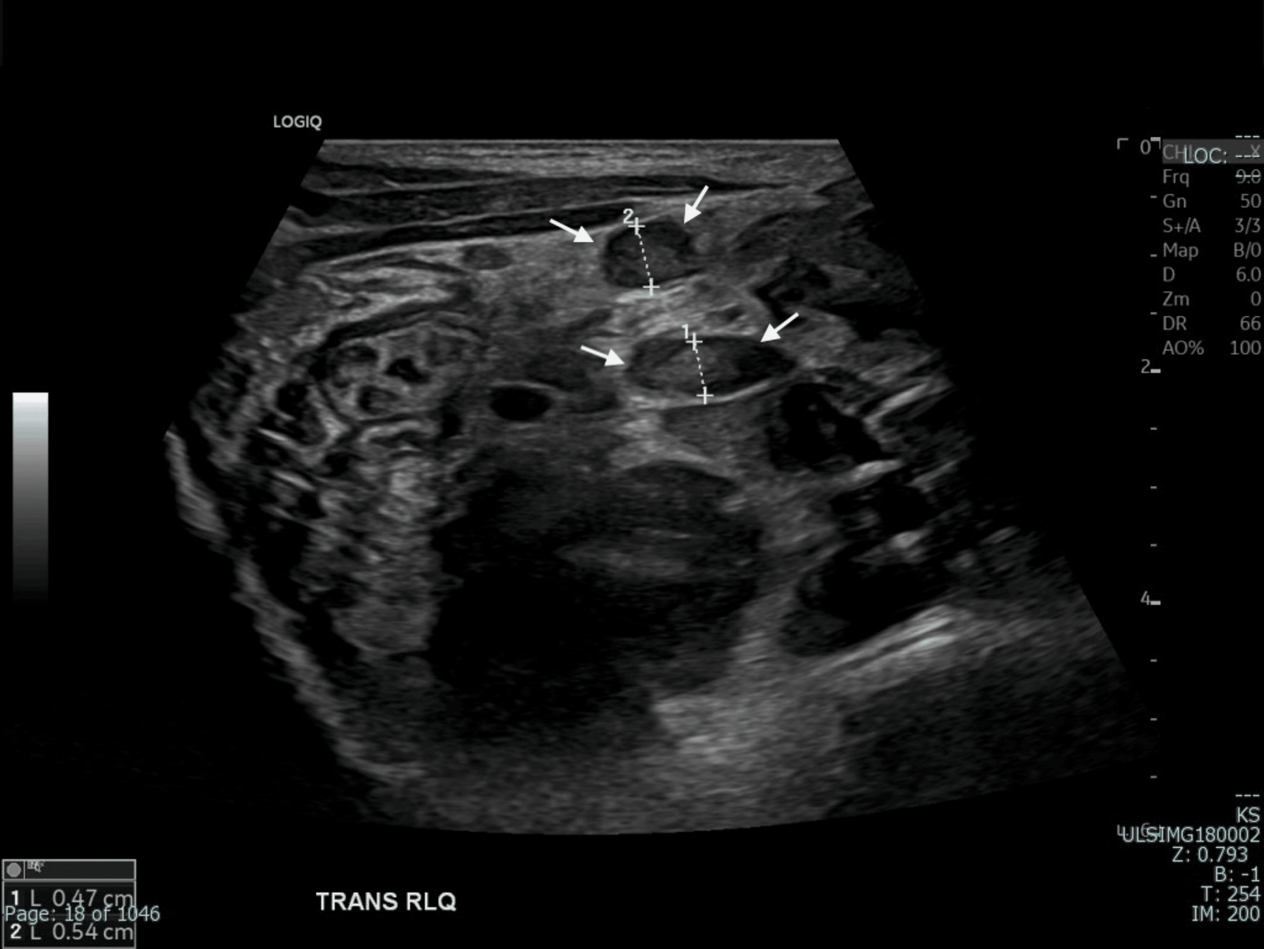
Enlarged mesenteric lymph nodes in the right lower quadrant, with the largest measuring up to 0.5 cm in short axis (white arrows).

The initial work-up, including laboratory tests and abdominal radiograph, showed no abnormalities. The respiratory viral panel was negative, and the urinalysis indicated no signs of a urinary tract infection. An abdominal ultrasound revealed the classic "target lesion" sign of intussusception, extending to the hepatic flexure with a maximum dimension of 4.5 × 3.3 cm. Based on its size and location, the intussusception was classified as ileocolic. Additionally, enlarged mesenteric lymph nodes were noted. A pelvic ultrasound demonstrated an enlarged non-compressible appendix, measuring 10 mm in transverse diameter, with increased vascularity of the appendiceal walls on color Doppler. No free fluid was detected (Figures 1-5).

**Figure 6:**
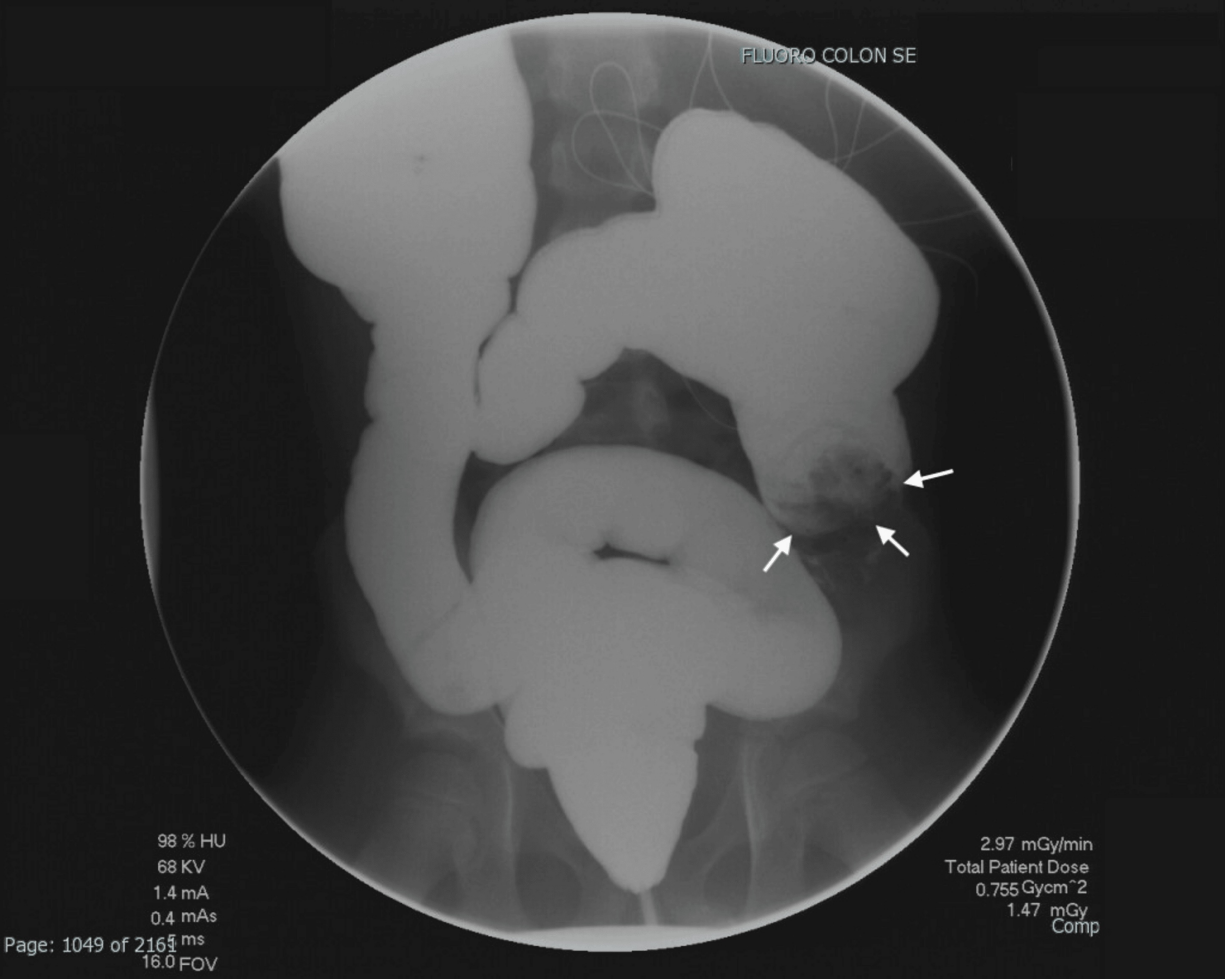
Water-soluble contrast enema reveals an intussusception at the ascending colon while the patient is in the prone position (white arrows).

On the same day of arrival, the patient underwent successful fluoroscopic hydrostatic reduction with water-soluble contrast enema (Figure 6). However, by the next day, the patient experienced increased abdominal discomfort. A repeat ultrasound revealed recurrent intussusception and a continued thickening of the appendix, measuring up to 8 mm, and remaining non-compressible. Surgical intervention was then decided upon.

**Figure 7:**
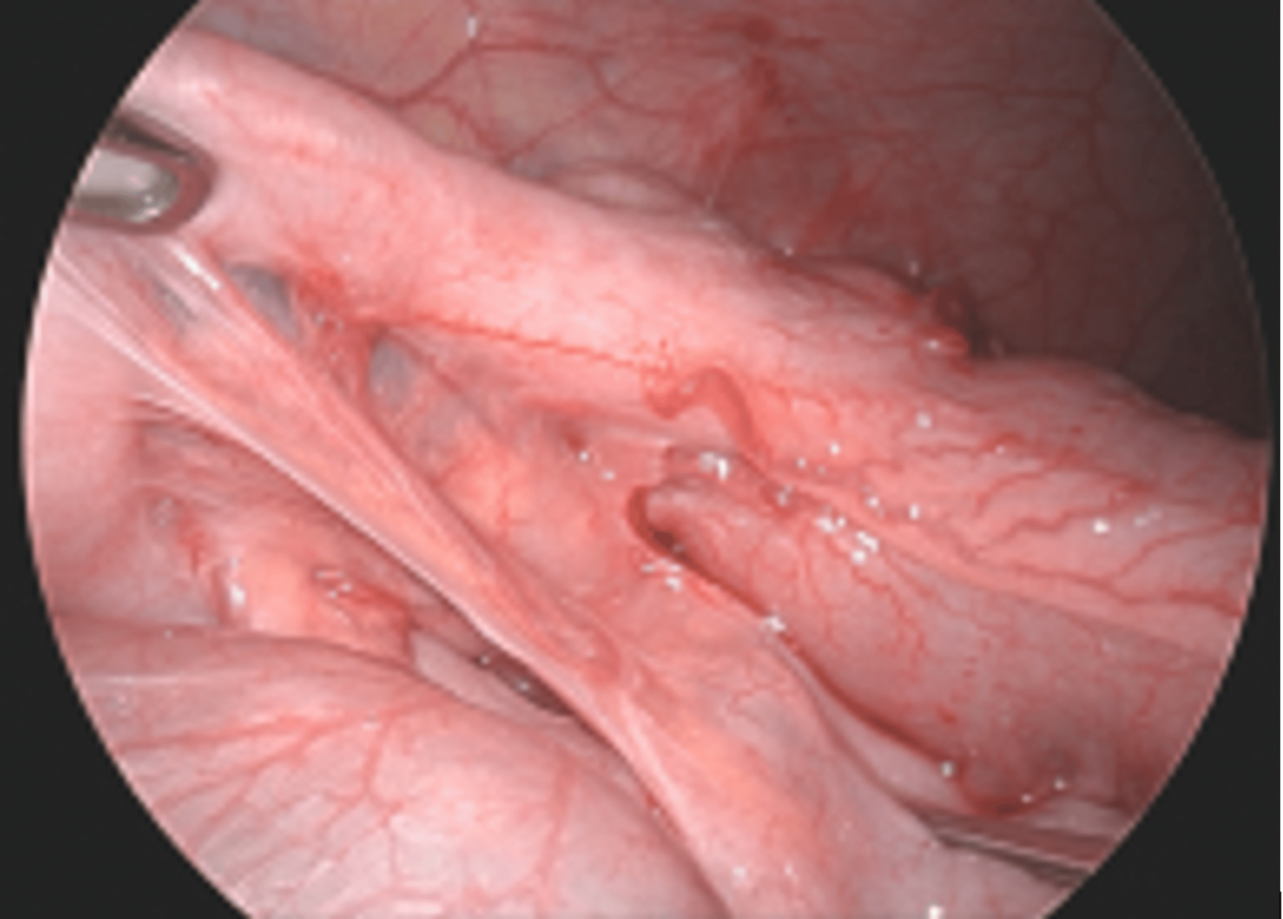
Prominent mesoappendiceal curtain with a turgid, inflamed appendix.

**Figure 8:**
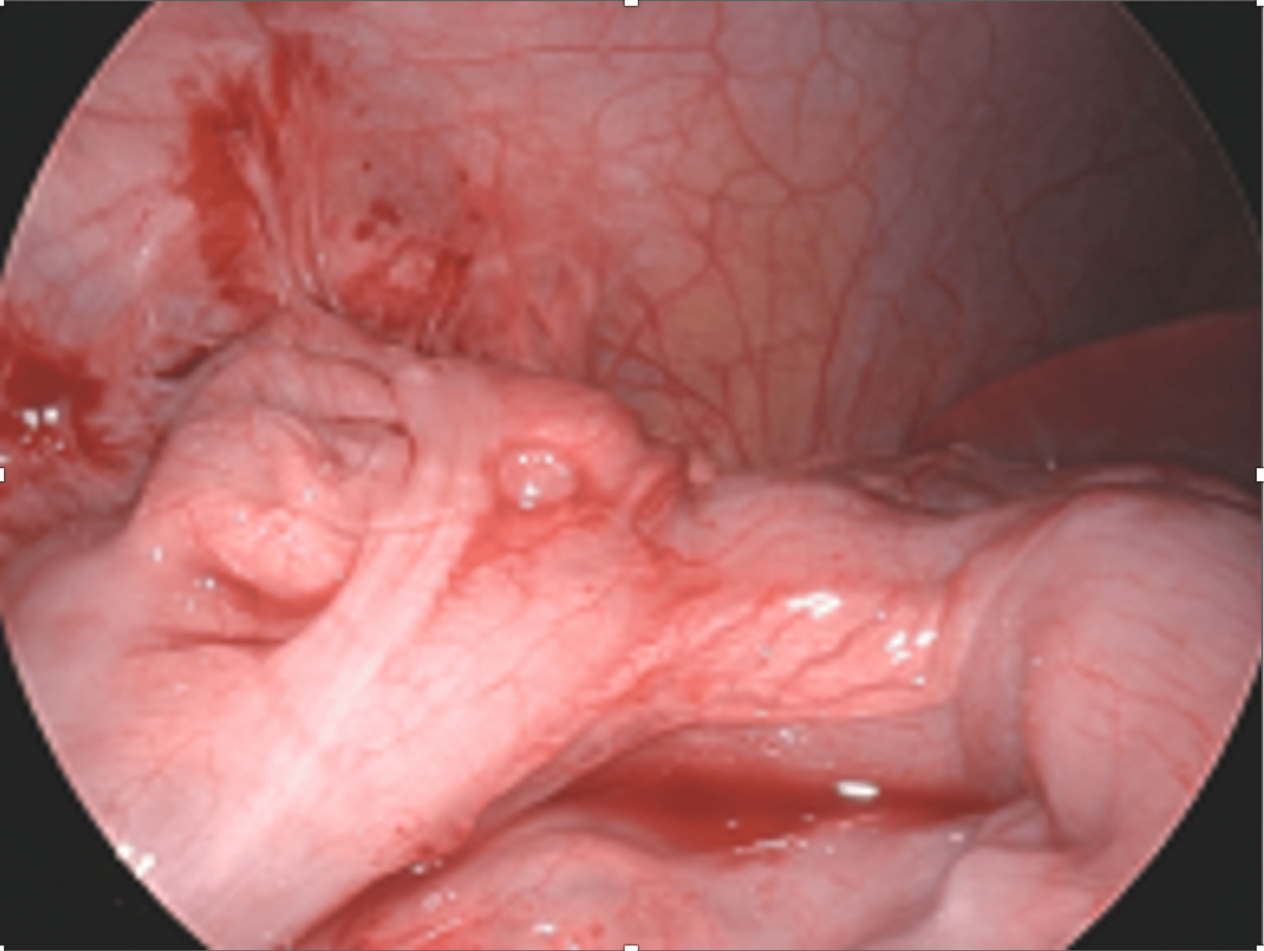
Mesoappendiceal curtain creating a cavern between the ileum and cecum.

**Figure 9:**
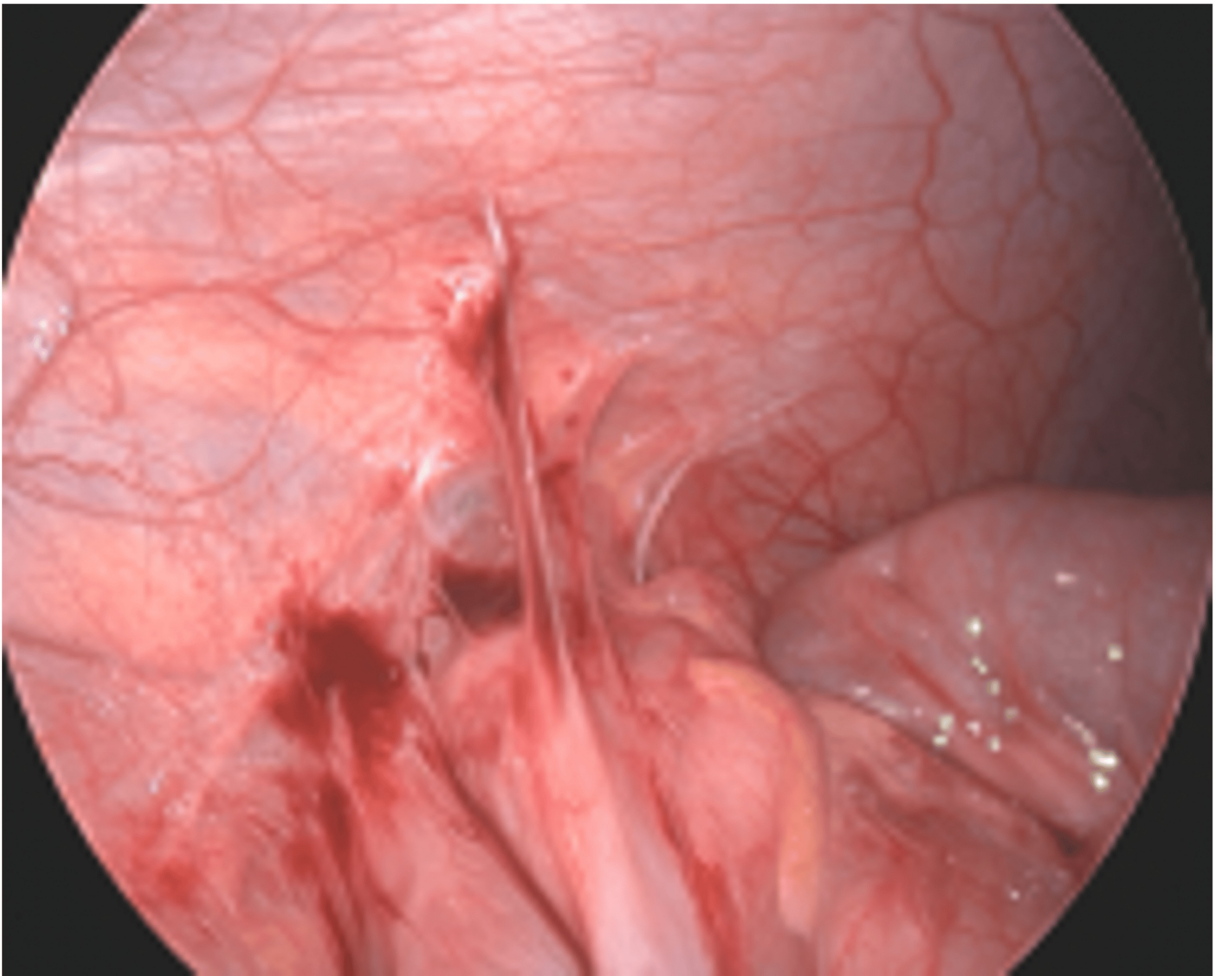
Inflammatory adhesions between the right colon and the peritoneal wall.

Intraoperative findings revealed a mesoappendix draping between the ileum and cecum, creating a potential cavern into which the ileum could telescope. Additionally, an inflammatory adhesion was found between the right colon and the peritoneal wall (Figures 7-9). This may have contributed to tethering the colon and promoting recurrent intussusception.

The patient's postoperative course was uncomplicated, and he was discharged on postoperative day 2.

## Discussion

Intussusception is commonly described as the invagination or telescoping of bowel segments, often leading to bowel obstruction, particularly in the small intestine. In addition to obstruction, severe complications such as bowel perforation or peritonitis can arise. Intussusception occurs more frequently in children than adults and is more common in males than females. The cause remains idiopathic in up to 90% of pediatric cases, with no identifiable source [3].

Several theories have been proposed regarding the etiology of intussusception. A recent history of infection has been strongly linked to increased cases, particularly in pediatric patients. Enlarged lymph nodes, specifically Peyer's patches, in the setting of infection, are believed to contribute to the development of intussusception. Common bacterial infections such as *Salmonella*, *Escherichia coli*, *Shigella*, and *Campylobacter* have been associated with an increased risk, as has viral gastroenteritis. Furthermore, in approximately one-third of pediatric cases, illnesses such as upper respiratory infections and otitis media precede the onset of intussusception. In younger children, particularly those under two years of age, adenovirus and human herpesvirus 6 have been identified as potential viral triggers [3].

In addition to infections, certain underdeveloped anatomical structures in children may predispose them to intussusception. For example, smaller taeniae coli and decreased rigidity in the cecum can play a role, as can immature longitudinal musculature near the ileocecal valve [4].

Intussusception in pediatric patients is often idiopathic, with no identifiable lead point in the majority of cases. However, when a lead point is present, it serves as a focal area for the bowel to telescope inward, causing the condition. Anatomic lead points, which act as focal areas that pull the peristalsing distal bowel into the proximal portion, are also considered in some cases. While malignancies are the most common lead points in adults, often requiring surgical intervention, congenital variants such as Meckel’s diverticulum are more typical in children [5]. Other lead points in pediatric intussusception include intestinal polyps (such as those associated with Peutz-Jeghers syndrome), duplication cysts, submucosal hematomas (seen in conditions like Henoch-Schönlein purpura), appendicitis, lipomas, hemangiomas, and occasionally tumors like lymphoma [5]. Recognizing the potential role of lead points is crucial in evaluating cases of pediatric intussusception, although idiopathic cases remain the most prevalent.

In this case, presumably, a prominent mesoappendiceal curtain appeared to create a cavern between the ileum and cecum, with adhesions involving an inverted, inflamed appendix and mesenteric fat. This likely acted as the primary factor, drawing the terminal ileum into the intussuscipiens.

Initially, the intussusception was thought to be idiopathic, but it recurred twice during hospitalization, despite a successful initial hydrostatic reduction. An inflamed appendix was noted - an uncommon coexistence of intussusception and appendicitis - but the exact relationship between the two remains unclear [5]. It is uncertain whether the intussusception led to strangulation and subsequent inflammation of the appendix. Notably, the inflamed appendix was not fully invaginated but was partially entrapped within the intussusception. We propose that the prominent mesoappendix was likely the cause of the multiple prior episodes, although the appendix itself was observed to be inflamed during the most recent recurrence.

## Conclusions

In summarizing this case, it's crucial to recognize that repeated instances of intussusception in children may arise from anatomical variations that act as potential lead points. While determining an exact cause for the recurring acute intussusception in this case was challenging, it's essential to consider a range of contributing factors. The possibilities, whether they involve prominent mesenteric lymph nodes due to acute appendicitis, a recent respiratory infection, or an unusual anatomical presentation of the mesoappendiceal curtain as a lead point, all potential etiologies must be thoroughly investigated in pediatric patients. Particularly in those with a history of multiple intussusception episodes, a comprehensive evaluation of all contributing factors is recommended to prevent further occurrences.
